# Yiqi Huoxue recipe ameliorates diabetic nephropathy by mediating VAPB–PTPIP51 complex to activate autophagy and regulate MAM contact

**DOI:** 10.3389/fnut.2025.1634555

**Published:** 2025-11-03

**Authors:** Linghao Dai, Mengyi Wang, Bo Wang, Haowei Liang, Yang Guan, Zhongyan Du, Hui Wang

**Affiliations:** ^1^Jinhua Academy, Zhejiang Chinese Medical University, Jinhua, China; ^2^School of Basic Medical Science, Key Laboratory of Blood-Stasis-Toxin Syndrome of Zhejiang Province, Zhejiang Chinese Medical University, Hangzhou, China; ^3^Academy of Chinese Medical Sciences, Zhejiang Chinese Medical University, Hangzhou, China; ^4^Fenghua District Traditional Chinese Medicine Hospital of Ningbo, Ningbo, China; ^5^School of Basic Medical Science, Zhejiang Key Laboratory of Blood-Stasis-Toxin Syndrome, Zhejiang Engineering Research Center for "Preventive Treatment" Smart Health of Traditional Chinese Medicine, Zhejiang Chinese Medical University, Hangzhou, China; ^6^Second Clinical Medical School, Zhejiang Chinese Medical University, Hangzhou, China; ^7^School of Pharmaceutical Sciences, Zhejiang Chinese Medical University, Hangzhou, China

**Keywords:** diabetic, nephropathy, Yiqi, Huoxue, recipe, autophagy, VAPB–PTPIP51, mitochondria-associated endoplasmic reticulum membranes

## Abstract

**Background:**

Diabetic nephropathy (DN), a major complication of diabetes mellitus (DM), poses a high mortality and a global health burden. Mitochondria-associated endoplasmic reticulum membranes (MAMs) and mediated autophagy are regarded as the crucial factors in the development of DN. The Yiqi Huoxue recipe (YHR), a traditional Chinese medicine formula, has been reported to treat DN and regulate autophagy, while its underlying mechanism remains unclear.

**Methods:**

Firstly, UPLC-MS/MS analysis was performed to identify the chemical components of YHR. Then, C57BL/6 J mice were injected with streptozotocin and fed with high-fat diet to induce DN. YHR (7.8, 15.6 g/kg/d) was administered via intragastric gavage for 8 weeks. Biochemical parameters and oxidative stress indicators were measured; H&E, PAS, and immunohistochemistry staining of nephrin were performed. Mitochondrial Ca^2+^ levels were assessed by flow cytometry, while autophagosomes and MAMs were examined using transmission electron microscopy (TEM). The expression levels of VAPB, PTPIP51, LC3 II/I, P62 were detected by Western blot. Podocytes overexpressing PTPIP51 or VAPB were analyzed for cell activity using the CCK-8 assay, autophagy flux by TEM, and the expression of LC3 II/I and P62 by Western blot. In si-PTPIP51-transfected and high-glucose (HG)-stimulated podocytes, CCK-8 assay, PCR, TEM, and immunofluorescence staining were performed to detect the YHR-containing serum on cell activity, mtDNA, MAMs, autophagosomes and LC3 expression.

**Results:**

The chemical fingerprint of YHR was constructed and composed chemicals were identified. In DN mice, YHR treatment reduced the elevated fasting blood glucose (FBG), total cholesterol (TC), triglycerides (TG), blood urea nitrogen (BUN), serum creatinine (Scr), urinary albuminuria (ALB), and microscale albuminuria (MAU) levels. It also alleviated kidney and glomerulus damage, mitochondrial Ca^2+^, oxidative stress, MAM abnormal contact, and activated autophagy. The enhanced expression of MAM complex, VAPB–PTPIP51, were also inhibited in YHR-treated groups. The cell activity and autophagosome formation were significantly inhibited in podocytes overexpressing PTPIP51 (oe-PTPIP51) and VAPB (oe-VAPB). In contrast, in HG-podocytes, si-PTPIP51 promoted the cell activity, mtDNA copy number, MAM contact, autophagosomes formation and LC3 expression. More importantly, the addition of YHR-containing serum enhanced this effect.

**Conclusion:**

YHR may protect kidneys in DN by regulating the MAM complex VAPB–PTPIP51 to trigger autophagy, providing insights into TCM’s clinical application and DN drug development.

## 1 Introduction

Diabetes mellitus (DM) is one of the most significant chronic metabolic diseases worldwide, with its incidence rising due to major changes in the aging population, obesity prevalence, diet, and lifestyle. The Global Burden of Disease Study Organization has indicated that 529 million individuals were affected by DM globally in 2021, and this number is projected to reach 1.31 billion by 2050 (1). Notably, China has the highest prevalence of DM, with approximately 141 million patients aged 20–79 years in 2021, and the number continues to rise annually (2). Worsening, diabetic nephropathy (DN), a severe complication of DM, can affect the entire kidney, leading to irreversible renal function damage or failure (3–5) Diabetes Care reports that DN occurs in up to 40% of patients with DM, resulting in life-threatening end-stage renal disease (ESRD) (6, 7). Furthermore, the all-cause mortality rate patients with DN is approximately 30 times higher than that of DM patients without nephropathy (8).

Common treatments for DN include lifestyle interventions, drugs, dialysis therapy, etc. Drugs used in clinical practice for treating DN primarily aim to lower blood sugar levels to slow renal function damage, including biguanides, sulfonylureas, glinides, *α*-glucosidase inhibitors, and insulin (9). Despite these measures, many DN patients still progress to ESRD. Therefore, innovative drugs are urgently needed to enhance and broaden current DN treatment options. In response to challenges in Western medicine treatment of DN, researchers are increasingly emphasizing traditional Chinese medicine (TCM) treatments. Yiqi Huoxue recipe (YHR), a traditional Chinese medicine formula, has been reported to treat DN. Our previous study demonstrated that YHR could notably improve renal function in glucose- and insulin-injected GK rats, indicating its potential to treat DN (10). Additionally, study has revealed that the protective effect of YHR on DN was associated with the regulation of podocyte autophagy to promote the degradation of advanced glycation end products (11). However, the underlying mechanism requires further investigation.

Podocytes, an epithelial cell, contribute significantly to glomerular filtration barrier formation (12, 13). High-glucose stimulation in diabetes triggers changes in mTORC1, AMPK, and Sirt1 nutrient sensing pathways, thereby reducing podocyte autophagy. This abnormal reduction in autophagy leads to insufficient podocyte renewal, vacuolation and foot process fusion, increasing podocyte injury, causing abnormal changes in functions and morphological traits, and becoming a crucial event in DN (5, 14, 15). Autophagy, a vital cellular metabolic process, is essential for cell homeostasis, growth, differentiation, and self-renewal. Research indicates that augmenting autophagy can protect podocytes and mitigate kidney damage (16). Thus, regulating podocyte autophagy could be a pivotal strategy to alleviate podocyte injury and improve DN. As a key mediator linking mitochondria and endoplasmic reticulum, mitochondria-associated endoplasmic reticulum membranes (MAMs) and their protein complexes have also been found to regulate autophagy. MAMs are essential for autophagosome formation, can regulate mitophagy by regulating ATG14 and Beclin1, and alterations in MAMs can lead to autophagy-related disorders. Studies on the role of MAMs in DN have reported complex findings. Xue et al. (17) suggested that adequate MAM sites in podocytes have a protective role against DN. In contrast, Wang et al. (18) reported that increased MAMs in diabetic podocytes are correlated with podocyte damage and renal insufficiency. These discrepant results indicate that the stability of MAMs may be a critical factor, where reduced stability could lead to mitochondrial dysfunction and podocyte injury. VABP–PTPIP51, a MAM complex, is found to affect autophagy through regulating mitochondrial–endoplasmic reticulum contact and mitochondrial Ca^2+^ transport (19, 20). In addition, overexpression of VABP–PTPIP51 diminishes autophagosome formation through strengthening mitochondrial–ER contacts, while silencing the protein releases this contact and triggers autophagy (21). Molecular docking analysis in our previous study also demonstrated promising bindings of active chemicals of YHR and VABP–PTPIP51 complex.

Consequently, we propose that YHR may regulate cell autophagy function by interacting with the MAM complex VAPB–PTPIP51 to ameliorate podocyte injury and ultimately achieve therapeutic effects for DN. This project aims to construct high-sugar podocyte model and mouse DN model to investigate the regulatory role of the MAM complex VAPB–PTPIP51 in podocytes and to determine whether YHR improves cell autophagy through targeting this process to improve DN. This project aims to elucidate the mechanism of YHR’s treatment of DN, providing theoretical research basis for the clinical application of traditional Chinese medicine in treating DN.

## 2 Materials and methods

### 2.1 Preparation of aqueous extract of YHR

Composition of the YHR: Raw *Astragalus Propinquus* 24.0 g, *Chuanxiong Rhizoma* 6.0 g, raw *Rehmannia Radix* 12.0 g, *Radix Puerariae* 12.0 g, *Radix Trichosanthis* 6.0 g, with a total of 60 g. YJR (600.0 g) was obtained from the TCM pharmacy of our hospital and soaked in distilled water at room temperature 25 °C for 1 h, then decocted 10 times the amount of water, filtered, and then distilled water is added and decocted again. The filtrates obtained from two extractions were combined and concentrated using a rotary evaporator at 80 rpm at 60 °C. The obtained aqueous extract of YHR, at a concentration of 2 g/mL, was then prepared as a lyophilized powder for storage.

### 2.2 Preparation of YHR-containing serum

The male SPF Sprague–Dawley (SD) rats, aged 8 weeks, were divided into two groups—blank and YHR—with five rats in each group. The YHR group received the drug at a dose of 10.8 g/kg via gastric intubation, three times daily for 3 days, while the control group was given pure water instead. After a 12-h fast, the rats received a final dose and were euthanized an hour later under CO_2_ anesthesia. Blood was collected from both groups, and serum was isolated and labelled as blank serum and YHR serum, respectively. The serum samples were pooled, inactivated at 56 °C for 30 min, filtered, and stored at −20 °C for *in vitro* experiments.

### 2.3 UPLC-MS/MS analysis of YHR

The chemical components of YHR were analyzed using SYNAPT G2-Si UPLC-Q-TOF/MS system (Waters), equipped with CORTECS® UPLC®T3 column (2.1 × 100 mm, 1.6 m, Waters). The mobile phase was 0.1% formic acid in purified water and acetonitrile at a flow rate of 0.3 mL/mL with an injection volume of 2 uL. An electrospray ESI ion source was used, which was scanned in positive and negative ion modes (50–1,200 m/z), respectively. The content of the main components in the aqueous extract was then quantified by measuring the chromatographic peak areas of the corresponding compounds at each concentration. The method was validated for specificity, linearity, precision, repeatability, stability, and recovery.

### 2.4 Animals and DN modeling

Male C57BL/6 J mice (17-20 g) were housed in an specific pathogen-free (SPF) environment with ad libitum dietary intake of water and 12-h cyclic confinement and darkness. After 1 week of acclimatization feeding, the mice were randomly assigned to either the type 2 diabetes model group and control group. The mice in the control group were fed a regular diet, while type 2 DM was modeled by administering a high-fat diet (HFD, 35% fat, 26% carbohydrate, 26% protein) for 6 weeks. At the end of week 6, all mice were fasted for 12 h. To induce the Type 2 Diabetes Mellitus (T2DM) model, Streptozotocin (STZ) (45 mg/kg per injection), freshly prepared in 0.1 M sodium citrate buffer (Solarbio), was administered via intraperitoneal injection once per week for four consecutive weeks (22). Mice with fasting blood glucose FBG ≥ 11.1 mM at 3 and 7 days after the last streptozotocin (STZ) injection were considered type 2 DM model mice. Then two mice were randomly selected from each group and euthanatized to obtain the kidney tissue. Hematoxylin and Eosin (H&E) staining was then performed to confirm the establishment of DN model. Following confirmation, the remaining mice were retained, ensuring that there are at least 24 eligible model mice for further experiments.

### 2.5 Animal grouping

The DN model mice were subsequently randomly assigned (*n* = 6 per group) to four experimental groups: the DN group, the YHR-low group, the YHR-high group, and the positive control (metformin) group. YHR groups were intragastric gavaged with YHR at 7.8 g/kg/d for low group and 15.6 g/kg/d for high group, converted from clinical doses, respectively. Metformin group was treated with 0.20 g/kg/d of metformin in the distilled water. Control and DN groups were treated with equal volume of saline. Drug administration was continued for 8 weeks. HFD was given to mice throughout the experiment.

### 2.6 Cell culture, transfection, and grouping

Mouse renal podocyte MCP-5 cells (Cellverse Co., Ltd.) were cultured in DMEM low-glucose medium containing 10% fetal bovine serum (FBS) at 37 °C in a 5% CO_2_ incubator.

About 1 × 10^5^ cells were seeded into a 24-well culture plate, and cell transfection was performed when the cells reached 50%–70% confluence. Over-expression PTPIP51 (oe-PTPIP51), over-expression VAPB (oe-VAPB), and its negative control over-expression NC (oe-NC) were provided by Shanghai Genepharma co., Ltd. The oe-PTPIP51, oe-VAPB and oe-NC were transfected into MPC5 cells using the Lipofectamine 3000 transfection kit according to the manufacturer’s instructions. The transfection efficiency of the introduced gene PTPIP51 and VAPB was verified by qRT-PCR 24 h later. After that, cells were introduced into five groups: control group cells were cultured in DMEM containing 5.5 mmol/L glucose for 72 h; the high glucose (HG) group cells were cultured in DMEM containing 30 mmol/L glucose; the oe-NC group cells were transfected with oe-NC and cultured in HG medium for 72 h; the oe-PTPIP51 group cells were transfected with oe-PTPIP51 and cultured in HG medium for 72 h; and the oe-VAPB group cells were transfected with oe-PTPIP51 and oe-VAPB, and cultured in HG medium for 72 h.

About 4 × 10^5^ cells were seeded into a 12-well culture plate, and cell transfection was performed when the cells reached 70%–80% confluence. PTPIP51 siRNA (si-PTPIP51) and its negative control siRNA NC (si-NC) were provided by GenePharma. The si-PTPIP51 and si-NC were transfected into MPC5 cells using the Lipofectamine 3000 transfection kit according to the manufacturer’s instructions. The transfection efficiency of the introduced gene PTPIP51 was verified by qRT-PCR 24 h later. After that, cells were introduced into five groups: Control group cells were cultured in DMEM containing 5.5 mmol/L glucose for 72 h; the HG group cells were cultured in DMEM containing 30 mmol/L glucose; the si-NC group cells were transfected with si-NC and cultured in HG medium for 72 h; the si-PTPIP51 group cells were transfected with si-PTPIP51 and cultured in HG medium containing 10% blank serum for 72 h; the si-PTPIP51 + YHR group cells were transfected with si-PTPIP51, and cultured in HG medium containing 10% YHR-containing serum for 72 h (23).

### 2.7 Kidney organ index and biochemical indicators detection

Animals were monitored weekly for body weight and 12-h fasting blood glucose (FBG). Urine was collected through a 24-h metabolic cage after last drug administration, and blood was taken from the orbits of the mice, centrifugated to obtain serum. After that, mice were euthanized using CO_2_, their abdomen was opened and kidneys were removed and weighted. Kidney organ index (%) was calculated using the formula: 100* kidney weight/ body weight. Then serum and urine samples were used to determinate of FBG, total cholesterol (TC), triglycerides (TG), blood urea nitrogen (BUN), serum creatinine (Scr), urinary albuminuria (ALB), and microscale albuminuria (MAU) levels with a fully automated biochemical tester (Hitachi 3,110, Hitachi). The levels of malondialdehyde (MDA), the activity of CAT, SOD, and GSH-PX in kidney tissues were calculated using the relevant ELISA kits (NanJing JianCheng Bioengineering Institute) according to the manufactures’ instructions with a microplate reader (CMaxPlus, MD).

### 2.8 H&E staining

Kidney tissues were fixed in 4% paraformaldehyde for 72 h. Subsequently, they underwent a series of treatments including dehydration, paraffin embedding, and sectioning into 5 μm sections. Kidney tissue sections were first deparaffinized by immersion in xylene followed by hydration through a gradient series of ethanol to distilled water. The sections were first stained with hematoxylin (Sigma) for 10 min, rinsed, and then stained with eosin (Sigma) for 30 s. After sealing the sections, the sections were observed under the microscope (Eclipse Ci-L, Nikon), and the results were recorded. Finally, the mean glomerular area was also calculated among the groups.

### 2.9 PAS staining

Paraffin sections were baked for 1 h, then deparaffinized and hydrated. The sections were then stained with periodic acid, washed, and stained with Schiff’s liquid (Ebiogo), and the nuclei were stained with Harry’s hematoxylin for 2 min. Finally, the results of pathological changes in the kidneys of the mice of each group were observed by light microscopy after dehydration, transparency, and sealing. The PAS-positive components—mainly glycogen, fibronectin, collagen fibers—were reddish-purple, and the nuclei showed blue color. A total of 20 glomeruli in each sample was scored to determine the severity of lesion, grading from 0 to 4, according to the percentage of glomerulosclerosis. Then the glomerulosclerotic index (GSI) of the sample was obtained based on the average score of these 20 glomeruli (24).

### 2.10 Immunohistochemistry staining

Following deparaffinization of mouse kidney tissue sections, citrate repair solution was used to repair the antigen; then endogenous peroxidase was blocked with blocking agent; the sections were closed with sealing solution for 30 min; and the primary antibody anti-nephrin (abcam, ab216341, 1:2000) was added and incubated at 4 °C overnight. On the next day, the sections were rewarmed at 37 °C for 1 h, rinsed with running water, incubated with secondary antibody, and rinsed with running water after incubation at 37 °C for 1 h. The sections were then subjected to 3,3′-Diaminobenzidine (DAB) color development. The sections were stained with DAB and hematoxylin, then dehydrated and sealed, and the images were captured and processed by K-Viewer pathology analysis software and Image-Pro Plus 6.0 management system for semi-quantitative analysis.

### 2.11 Mitochondrial Ca^2+^ detection by flow cytometry

Kidney tissue was minced and digested using pancreatic enzymes. The supernatant was discarded after centrifugation at 600 g at room temperature for 5 min. Cells were resuspended in Rhod-2 staining solution (Beyotime Biotechnology) to achieve a cell density of 2 × 10^6^ cells/mL. The suspension was incubated at 37 °C for 30 min. Subsequently, the suspension was centrifuged at 600 g at 4 °C for 3 min to pellet the cells, and the supernatant was discarded. After washing with phosphate-buffered saline (PBS), repeat the centrifugation procedure and discard the supernatant. Add an appropriate amount of PBS to the pellet and analyze using a flow cytometer (Novocyte, Agilent).

### 2.12 Quantitative real-time PCR

Total RNA was isolated from cultured cells and extracted using the EZ-10 Spin Column Total RNA Isolation Kit (BBI). RNA was subjected to reverse transcription reaction using TRUEscript RT MasterMix (OneStep gDNA Removal) (Aidlab Biotechnologies Co., Ltd) according to the manufacturer’s specifications. Primers were synthesized by Sangon Biotech, and the primer sequences are shown in Supplementary Table S2.

### 2.13 Western blot assay

Kidney tissues were cut into shreds, incubated with Lysis Buffer on ice to generate lysate, and then centrifuged to extract the supernatant protein. The concentration of total protein was detected using a BCA kit (Beyotime Biotechnology). About 50 μg sample was loaded onto SDS-PAGE gels for electrophoresis and transferred to a PVDF membrane. After blocking with non-fat milk solution, primary antibodies—anti-LC3, anti-P62, anti-VAPB, and anti-PTPIP51 (1:1000, Affinity)—were added to the membrane for overnight, and secondary antibody-anti-rabbit IgG HRP-linked antibody was added for 1 h incubation. Following incubation, the membrane was washed with Tris-Buffered Saline with Tween 20 (TBST). Immunoreactive bands were developed by enhanced chemiluminescence (ECL) reagents. The intensity of the bands was quantified using the Image J software.

### 2.14 CCK-8 assay

MCP-5 cells were treated accordingly, then medium was changed, and CCK-8 reagent (Beyotime Biotechnology) was added. The cells were incubated, OD values were measured using a microplate reader (CMaxPlus, MD), and the cell viability was calculated.

### 2.15 mtDNA detection

MCP-5 cells (1 × 10^6^–1 × 10^7^) were centrifuged to remove the supernatant, and then the cells were collected. The mtDNA was extracted from the mitochondria using the DNA extraction kit (Solarbio), and then the mtDNA was amplified by PCR amplifier. The amplified products were detected using 1.5% agarose gel electrophoresis and scanned with a gel imager to obtain the integrated optical density of each band and measure the copy number of mtDNA.

### 2.16 Immunofluorescence staining

Appropriate amount of cells were inoculated onto slides, treated with drugs, discarded from the culture solution, and fixed by 4% paraformaldehyde. The cells were then washed with PBS and added with 0.5% Triton X-10 to permeate the cell membrane. Afterwards, cells were rewashed with PBS; 3% BSA was added to seal the cells, and then the cells were incubated with primary antibody—anti-LC3A/B (1:200, affinity) antibody—and secondary antibody—anti-IgG H&L (1:500, Abcam) sequentially. After that, DAPI staining solution (Sigma) was added to stain the cell nuclei. Finally, the slides were sealed and placed under an inverted fluorescence microscope (Ts2-FC, Nikon) for observation.

### 2.17 Transmission electron microscopy

Firstly, kidney tissue or MCP-5 cells were fixed in 2.5% glutaraldehyde for 3 h. This was followed by washing with PBS and subsequent fixation in 1% osmium acid solution for 2 h. Afterward, the samples were washed again and dehydrated by gradient ethanol. Then, embedding agent/acetone mixture from V/V = 1/1, V/V = 3/1, to total embedding agent was used to embed the samples. The samples were heated overnight at 70 °C using a heated polymerizer (UVC3 Cryo Chamber, PELCO) and were cut into sections (70 nm). The sections were further stained by 3% uranyl acetate–lead citrate solution for 30 min and were observed under TEM (H7650, Hitachi).

### 2.18 MAM contact levels detection by TEM

The MAMs contact sites were defined as the regions where the distance between the endoplasmic reticulum (ER) and the mitochondrial outer membrane was ≤50 nm. This criterion was applied to TEM images to quantify the extent of close ER–mitochondria contacts. The linear contact length between the ER and mitochondrial membranes was measured using Image J software. To eliminate potential bias arising from variations in mitochondrial size, this measured contact length was normalized to the perimeter of the corresponding mitochondrion.

### 2.19 Autophagy flux detection

The autophagy flux is commonly monitored using the GFP-LC3-RFP-LC3ΔG probe. This assay relies on the differential stability of the two tags: GFP-LC3 is degraded in autolysosomes, while RFP-LC3ΔG remains stable in the cytoplasm. Thus, autophagy flux can be quantified by comparing the signals from these two fluorescent reporters.

### 2.20 Statistical assay

Data are presented as means ± SD. The analysis was conducted using GraphPad Prism 8.02 (GraphPad Software, San Diego, CA) and SPSS 25.0 (SPSS, Chicago, IL, USA). The Shapiro–Wilk test and Levene’s test were performed to test the normality of the model residuals and the homogeneity of variance, respectively. Significant differences among multiple groups were examined using one-way ANOVA, followed by Tukey’s *post-hoc* test for uniform variance or Dunnett’s T3 test for non-uniform variance. *p* < 0.05 was considered statistically significant.

## 3 Results

### 3.1 The chemical fingerprint of YHR

**Figure 1:**
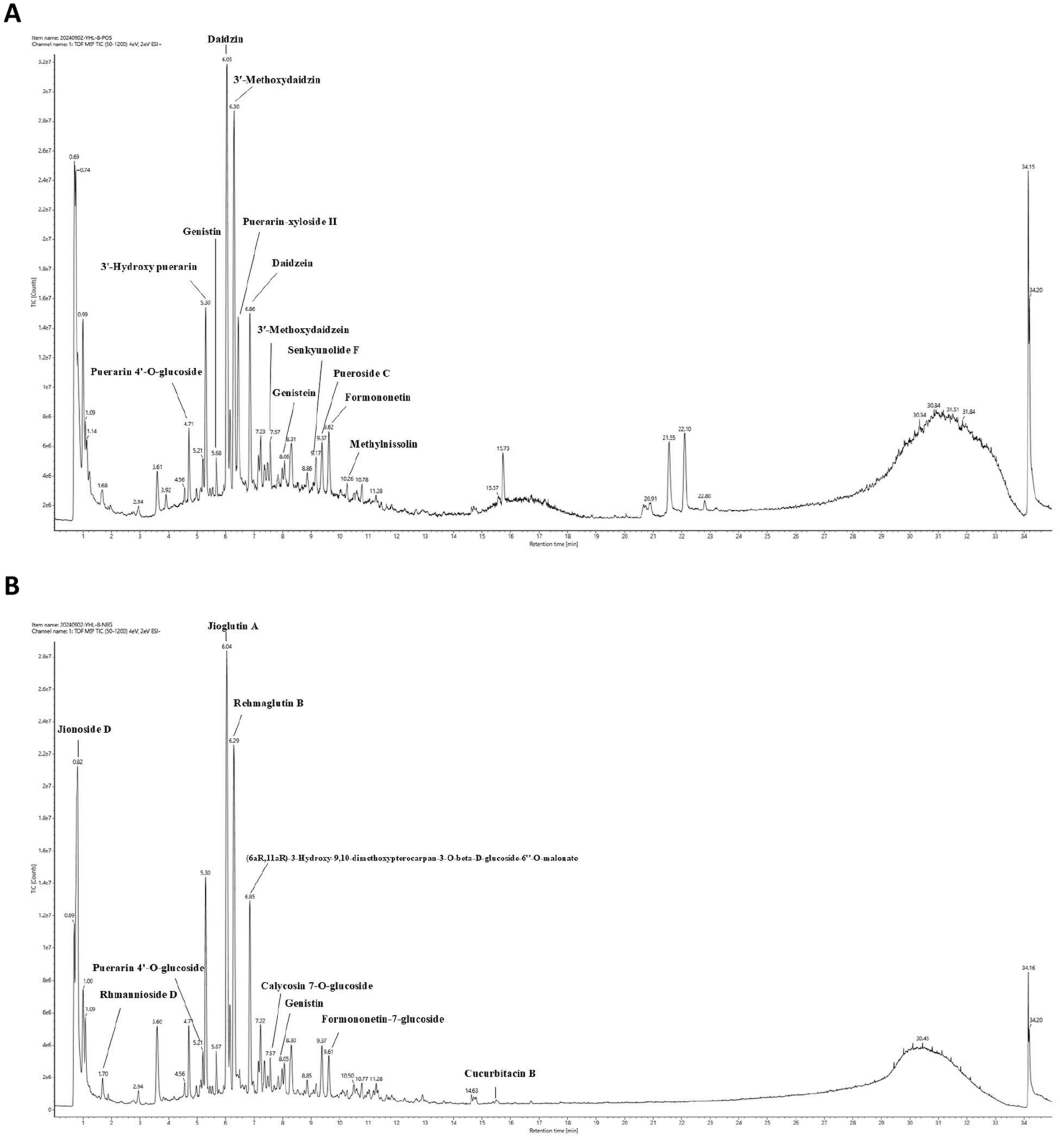
Chemical fingerprint construction of YHR by UPLC–MS/MS analysis. **(A)** The chemical fingerprint of YHR at positive mode; **(B)** The chemical fingerprint of YHR at negative mode. YHR: Yiqi Huoxue recipe.

To unravel the intricate chemical profile of the traditional Chinese medicine YHR, we employed UPLC–MS/MS analysis to establish its chemical fingerprint. This approach led to the identification of 44 chemicals in positive ion mode, 47 in negative ion mode (Figures 1A,B, Supplementary Table S1), with a total of 83 distinct compounds after eliminating redundancies. Notable among these were Daidzin, Jioglutin A, 3′-Methoxydaidzin, Puerarin-xyloside II, Genistein, Rehmaglutin B, etc., showcasing the rich diversity of YHR’s constituents.

### 3.2 Effect of YHR on biochemical indexes of DN mice

**Figure 2:**
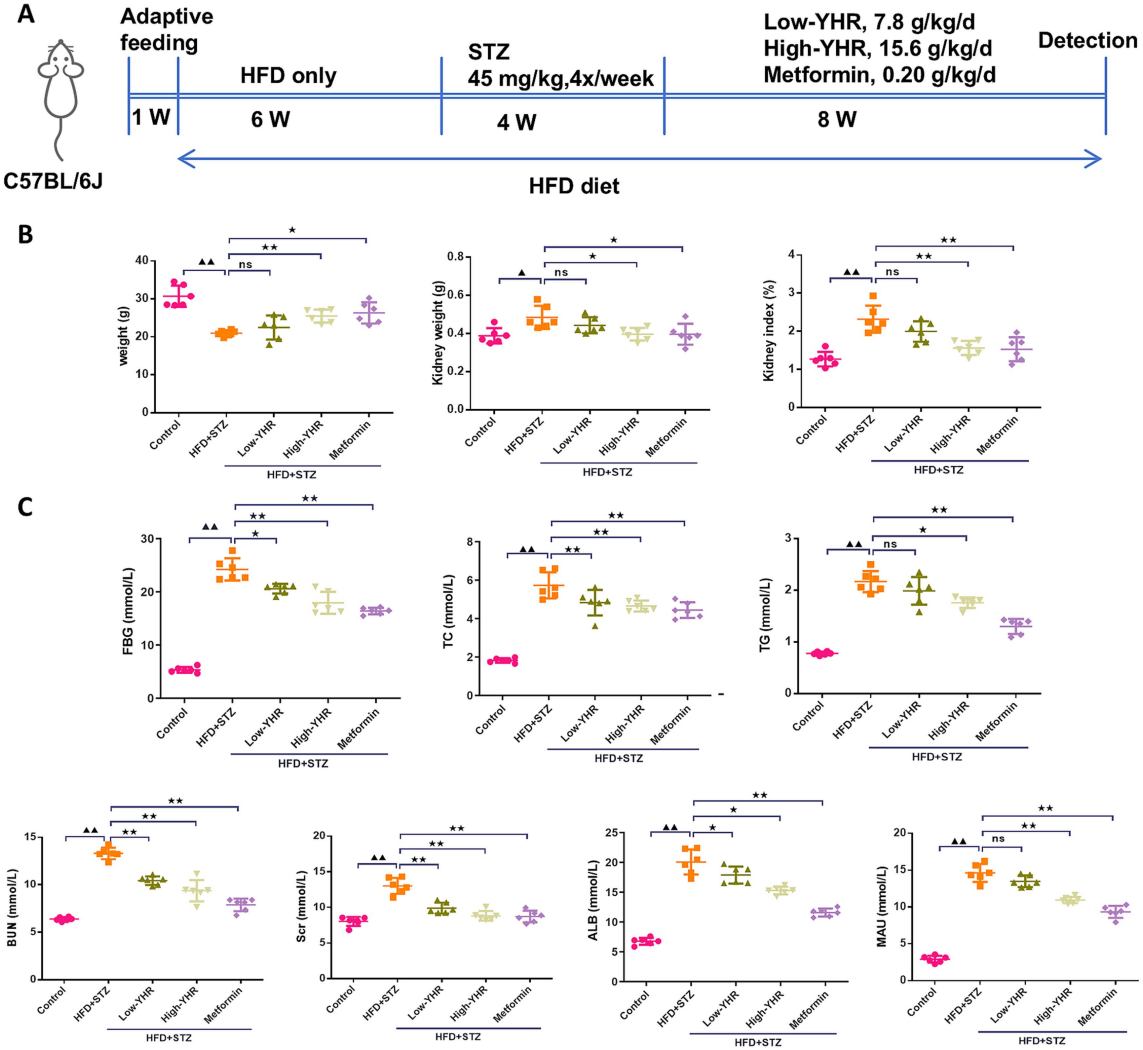
Detection of biochemical indexes of DN mice. C57BL/6 J mice were fed with high-fat diet and injected with streptozotocin to induce DN; then YHR at different dosages as well as positive control metformin were intragastric gavaged for 8 weeks. **(A)** Schematic of the study design; **(B)** body weight, kidney weight, and kidney organ index (%) of the mice were recorded and calculated. **(C)** FBG, TC, TG, BUN, Scr, ALB, and MAU levels were detected. DN, diabetic nephropathy; STZ, streptozotocin; HFD, high-fat diet; YHR, Yiqi Huoxue recipe; FBG, fasting blood glucose; ALB, urinary albuminuria; MAU, microscale albuminuria. Data were expressed as mean ± standard deviation, n = 6. Compared to the control group, ^▲^ *p* < 0.05, ^▲▲^ *p* < 0.01; compared to the HFD + STZ group, * *p* < 0.05, ** *p* < 0.01.

A combination of HFD and STZ injection was used to establish the animal model to investigate the therapeutic effect of YHR (Figure 2A). As shown in Figure 2B, after modeling, the body weight of mice was decreased compared to the normal control mice, while YHR administration at high dosage gained weight, so does metformin. Conversely, kidney weight and the organ index increased post modeling but were decreased by YHR administration at high-dosage level. Additionally, the levels of FBG, TC, TG, BUN, Scr, ALB, and MAU increased in the model group, and YHR treatment lowered these indexes, especially in high-dosage groups (Figure 2C).

### 3.3 YHR treatment improved the kidney injury of DN mice

**Figure 3:**
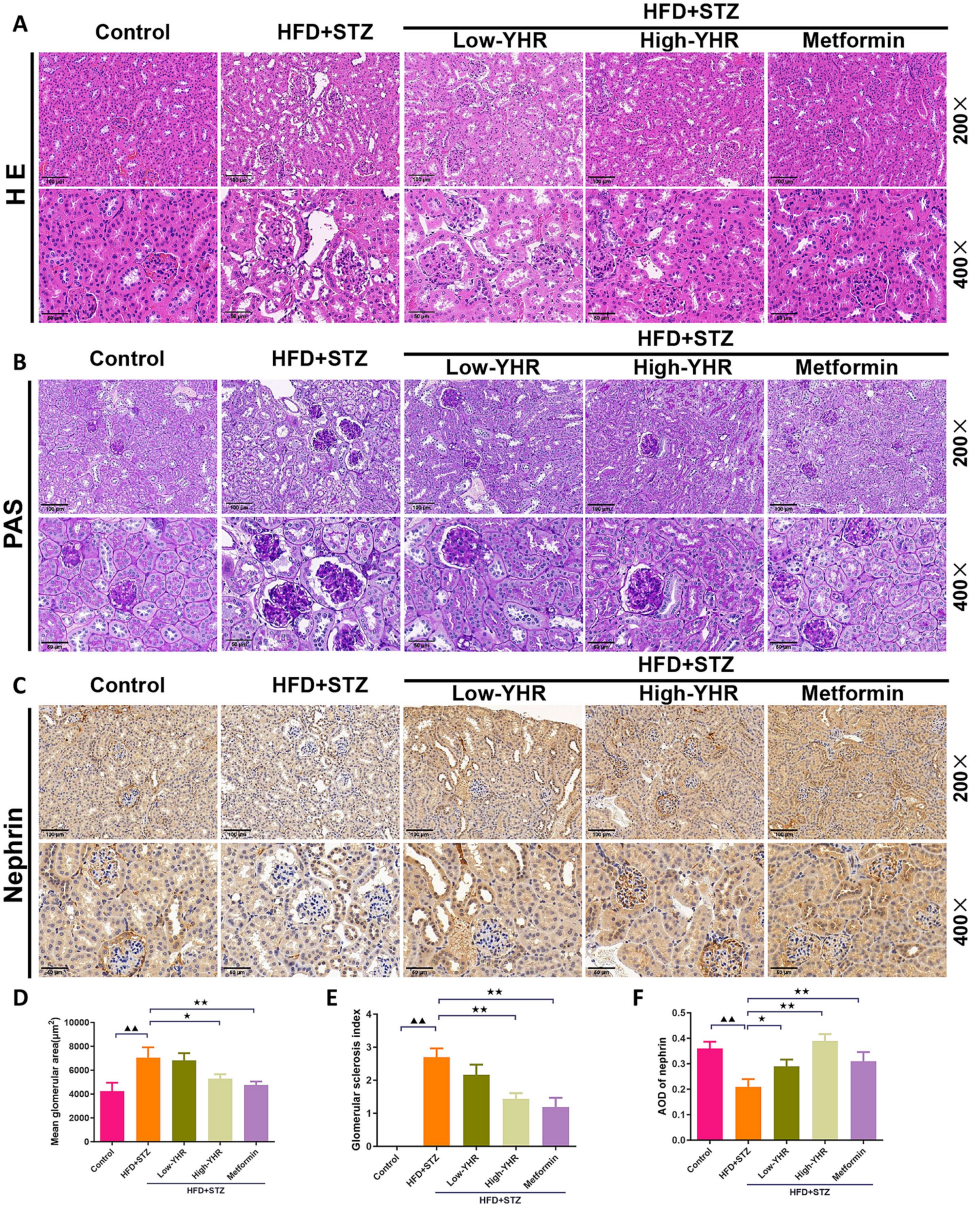
Evaluation of kidney pathologic impairment in DN mice. **(A)** H&E staining of the kidney tissues, **(B)** PAS staining of the kidney tissues, **(C)** immunohistochemistry staining of nephrin, a biomarker of podocytes. 200×, scale bar = 100 μm, 400, scale bar = 50 μm. **(D)** Mean area of glomerulus was also calculated based on H&E staining. **(E)** glomerulosclerotic index was calculated based on PAS staining. **(F)** The relative density of nephrin was quantified by calculating AOD value. DN, diabetic nephropathy; STZ, streptozotocin; HFD, high-fat diet; YHR, Yiqi Huoxue recipe; AOD, average optical density. *n* = 3. Compared to the control group, ^▲^ *p* < 0.05, ^▲▲^ *p* < 0.01; compared to the HFD + STZ group,* *p* < 0.05, ** *p* < 0.01.

H&E staining and periodic acid-Schiff (PAS) staining were used to assess the histopathologic changes of kidney in DN mice. As shown in Figures 3A,B,D,E, compared to the control mice, in the model group, the kidney tissue of mice was severely damaged, with glomerular atrophy, vacuolation of renal tubules, renal tissue thylakoid stroma hyperplasia, glomerular basement membrane thickening, enlarged balloon lumen. The glomerular area and glomerulosclerotic index also increased. Compared to the model group, the extent of kidney tissue damage in the YHR group was significantly improved, marked by a reduction in the degree of glomerular atrophy, basement membrane thickening, and tubular vacuolization. These findings were further accompanied by decreased glomerular areas and glomerulosclerotic indexes. In addition, the expression of nephrin, indicators of podocyte, was also detected in kidney tissues (Figures 3C,F). As a result, the model group showed less positive expression of nephrin compared to that in control group, and YHR treatment enhanced its expression in kidney.

### 3.4 YHR treatment alleviated the mitochondrial Ca^2+^ and oxidative stress in DN mice

**Figure 4:**
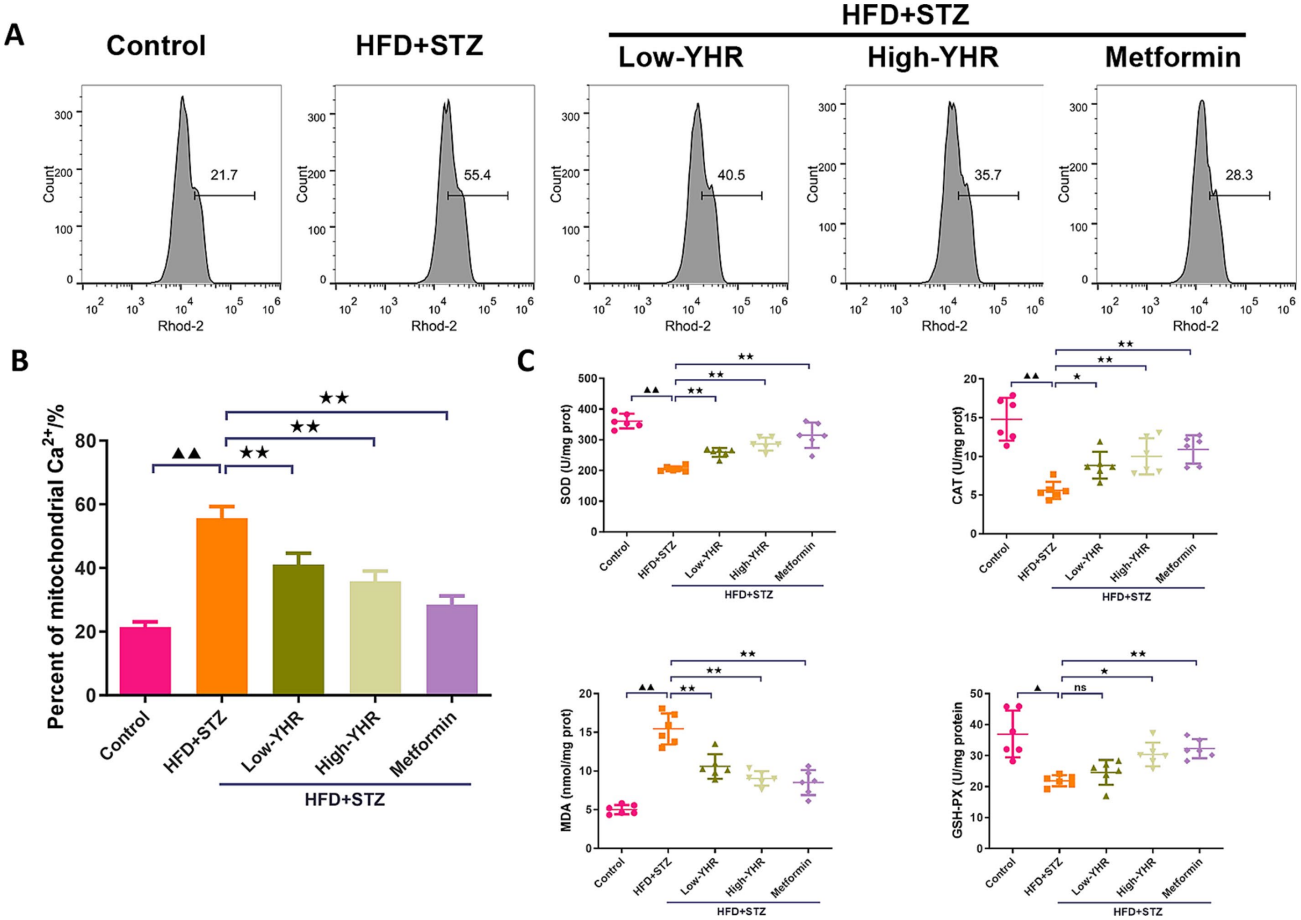
Detection of the mitochondrial Ca^2+^ and oxidative stress in DN mice. **(A,B)** Mitochondrial Ca^2+^ was detected by flow cytometry in kidney tissues. *n* = 3. **(C)** Oxidative stress indicators, SOD, CAT, MDA, and GSH-PX, were detected in kidney tissues. *n* = 6. DN, diabetic nephropathy; STZ, streptozotocin; HFD, high-fat diet; YHR, Yiqi Huoxue recipe. Data were expressed as mean ± standard deviation. Compared to the control group, ^▲^ *p* < 0.05, ^▲▲^ *p* < 0.01; compared to the HFD + STZ group,* *p* < 0.05, ** *p* < 0.01.

As the dysregulation of Ca2^+^ fluxes is involved in DN, the mitochondrial Ca^2+^ was detected. As shown in Figures 4A,B the percent of mitochondrial Ca2^+^ was dramatically increased in DN model group compared to that in control group. Following YHR treatment, the mitochondrial Ca2^+^ decreased both in low and high YHR groups. To evaluate the oxidative stress in DN mice, the SOD, CAT, and GSH-PX activities, MDA content in kidney tissues was determined after drug treatment. As shown in Figure 4C, the mice in the DN group were under oxidative stress status with increased MDA contents and suppressed SOD, CAT, and GSH-PX activities in kidney tissues in comparison with those in control group. After treatment, lower MDA contents and higher SOD, CAT, and GSH-PX activities in the kidney tissues of YHR groups were observed.

### 3.5 Effect of YHR on MAM contact and autophagy in kidney of DN mice

**Figure 5:**
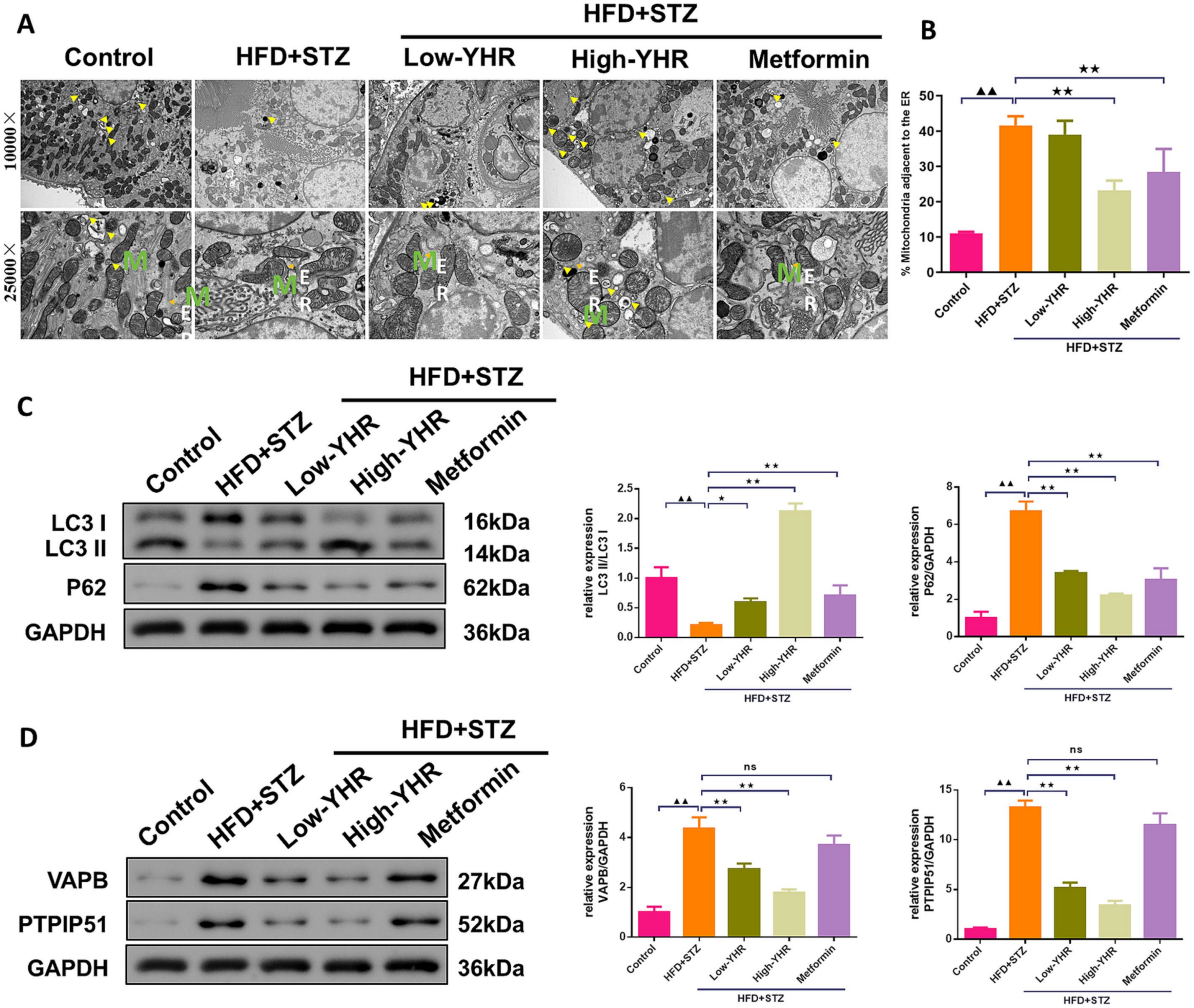
Evaluation of MAM changes and autophagy in kidney of DN mice. **(A)**TEM was used to observe the MAM contact and autophagosome in kidney tissues, yellow arrow indicates autophagosome, *M* indicates mitochondria, ER indicates endoplasmic reticulum. **(B)** The proportion of mitochondria adjacent to the ER in kidney tissue. *n* = 3. **(C)** The protein expression of autophagy indicators, LC3 II/I and p62, were detected by Western blot. **(D)** The protein expression of VAPB and PTPIP51 was detected by Western blot. Data were expressed as mean±standard deviation, *n* = 6. * *p* < 0.05, ** *p* < 0.01. TEM, transmission electron microscope; DN, diabetic nephropathy; STZ, streptozotocin; HFD, high-fat diet; YHR, Yiqi Huoxue recipe. Data were expressed as mean ± standard deviation, *n* = 3. Compared to the control group, ^▲^ *p* < 0.05, ^▲▲^ *p* < 0.01; compared to the HFD + STZ group, * *p* < 0.05, ** *p* < 0.01.

The MAM structure and autophagosome in kidney tissues were observed using TEM. The result indicated that the ultrastructure of kidney tissues underwent significant changes. Compared to the control group, the model group showed a significant decrease in cell autophagy. Also, compared to the control group, the model group showed a significant increase in ER–mitochondria contact levels, suggesting more compact MAM of kidney tissues in the model group. However, in the high-dose YHR group, kidney tissue autophagy was significantly enhanced and the MAM contact was relaxed compared to the model group (Figures 5A,B). The expression of autophagy-related proteins was also detected. The LC3 II/I ratio decreased, while p62 expression increased in model kidneys compared to those of controls (Figure 5C). The administration of YHR effectively counteracted these effects. In addition, the protein expressions of VAPB and PTPIP51 were significantly increased in the model group, which were decreased after YHR treatment (Figure 5D).

### 3.6 VAPB–PTPIP51 inhibited the autophagy of podocytes in HG condition

**Figure 6:**
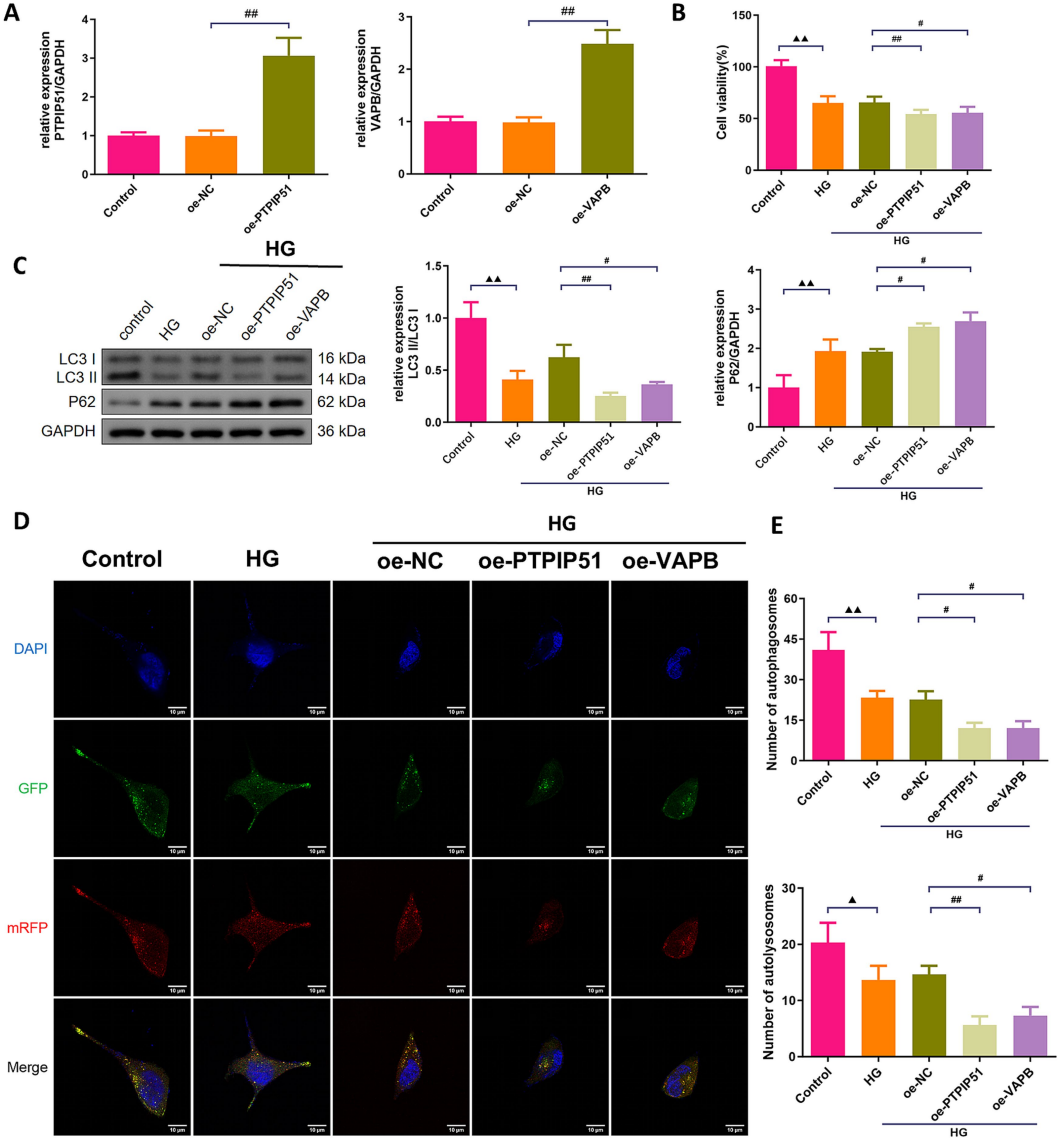
Overexpression of VAPB-PTPIP51 suppressed podocyte autophagy. MCP-5 cells were transfected with oe-PTPIP51, oe-VAPB or negative control (oe-NC), and stimulated with HG (50 mmol/L glucose). **(A)** The mRNA expressions of PTPIP51 and VAPB were detected by qRT-PCR. *n* = 3. **(B)** The cell activity was detected by CCK-8 assay. *n* = 6. **(C)** The protein expression of autophagy indicators, LC3 II/I and p62, were detected by Western blot. **(D)** Changes in autophagosomes and autophagic lysosomes in MPC-5 cells. *n* = 3. **(E)** Changes in the number of autophagosomes and autophagic lysosomes in MPC-5 cells by subgroups. *n* = 3. HG: high glucose. Compared to the control group, ^▲^ *p* < 0.05, ^▲▲^ *p* < 0.01; compared to HG group, * *p* < 0.05, ** *p* < 0.01; compared to the oe-NC group, # *p* < 0.05, ## *p* < 0.01.

To confirm the functional roles of PTPIP51 and VAPB, we transfected MCP-5 cells with oe-PTPIP51, or oe-VAPB. Oe-PTPIP51 and oe-VAPB transfection significantly increased the corresponding mRNA levels (Figure 6A). Under HG conditions, cell viability was significantly reduced in groups treated with oe-PTPIP51 or oe-VAPB compared to the oe-NC (Figure 6B). Furthermore, analysis of autophagy-related proteins revealed that exposure to HG significantly decreased the LC3 II/I ratio and increased p62 expression compared to the control group. Additionally, under HG conditions, transfection with oe-PTPIP51 or oe-VAPB led to a more pronounced reduction in the LC3 II/I ratio and a greater elevation of p62 than observed in the oe-NC group (Figure 6C). Compared to the control group, the HG group showed a reduction in autophagosomes and autophagic lysosomes. This reduction was also evident in the oe-PTPIP51 and oe-VAPB groups as compared to the oe-NC group (Figures 6D,E).

### 3.7 YHR activated the autophagy of podocytes via inhibiting PTPIP51

**Figure 7:**
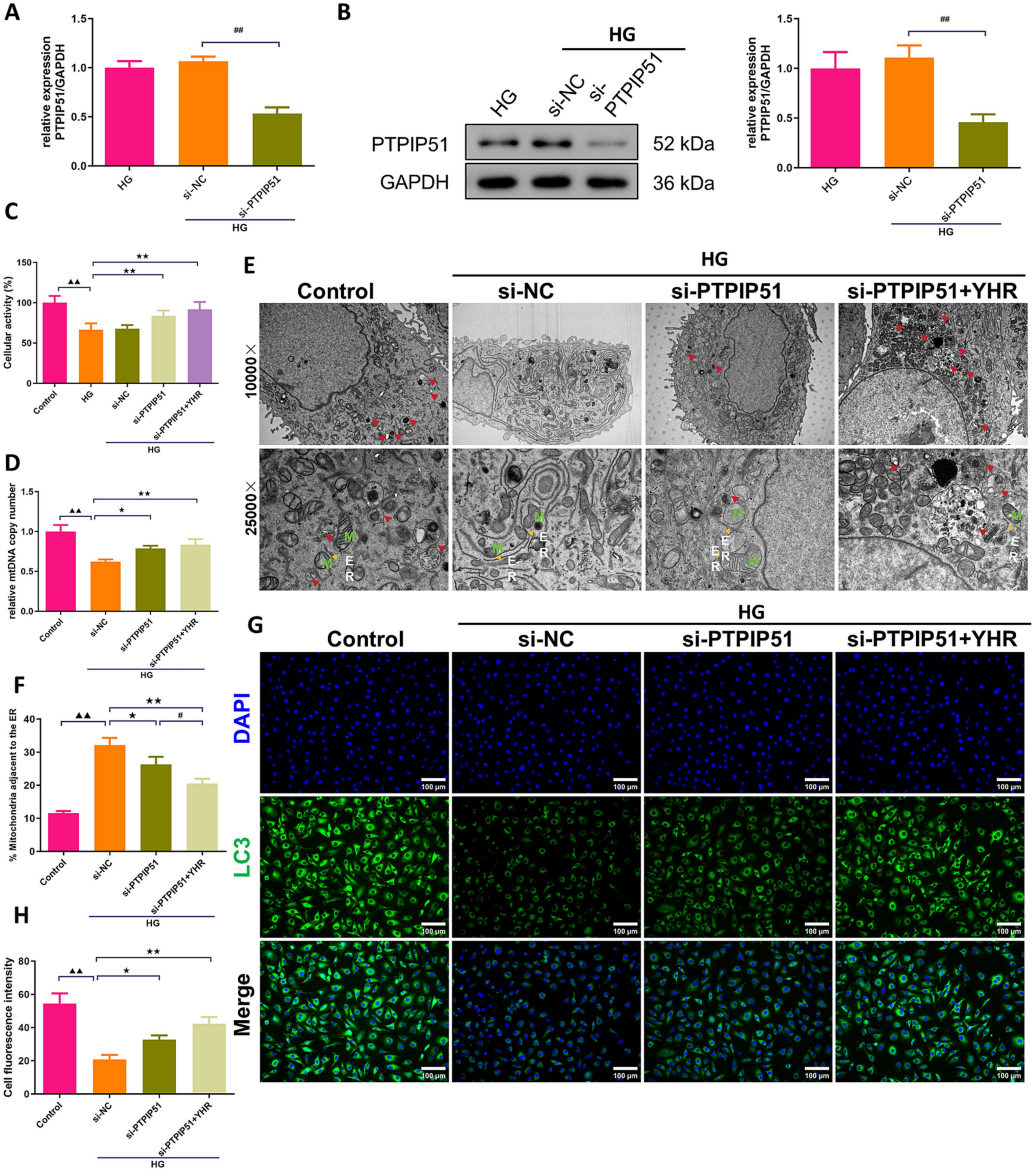
YHR activated the autophagy of podocyte via inhibiting VAPB–PTPIP51. Podocytes, MCP-5 cells, were transfected with si-PTPIP51 or negative control (si-NC) and stimulated with HG (50 mmol/L glucose), then 10% YHR-containing serum was added. **(A)** The mRNA expression of PTPIP51 was detected by qRT-PCR. *n* = 3. **(B)** The protein expression of PTPIP51 was detected by Western blot. *n* = 3. **(C)** The cell activity was detected by CCK-8 assay. *n* = 6. **(D)** mtDNA copy number was detected. *n* = 3. **(E)** TEM was used for MAM and autophagy detection, red arrow indicates autophagosome, *M* indicates mitochondria, ER indicates endoplasmic reticulum. **(F)** The proportion of mitochondria adjacent to the ER in MCP-5 cells. *n* = 3. **(G,H)** The expression of LC3, autophagy indicator, was detected by immunofluorescence staining. 200×, scale bar = 100 μm, 400, scale bar = 50 μm. *n* = 3. HG, high glucose; YHR, Yiqi Huoxue recipe. Data were expressed as mean ± standard deviation, *n* = 3. Compared to the control group, ^▲^ *p* < 0.05, ^▲▲^ *p* < 0.01; compared to the si-NC group, * *p* < 0.05, ** *p* < 0.01; compared to the HG group, * *p* < 0.05, ** *p* < 0.01; compared to the si-PTPIP51 group, # *p* < 0.05, ## *p* < 0.01.

In podocytes treated with HG for *in vitro* simulation of DN, we also investigate the effect of YHR on cell autophagy. MCP-5 cells were transfected with si-PTPIP51, and significant decreases in both mRNA and protein expression levels of PTPIP51 were confirmed (Figures 7A,B). Treatment with high glucose (HG) decreased the viability of podocytes, while si-PTPIP51 transfection and YHR addition restored the cell viability (Figure 7C). The mtDNA copy number was also decreased in the HG group but was increased by both si-PTPIP51 transfection and YHR treatment (Figure 7D). The microscopic state of the cell was also observed using TEM. It could be observed that revealed that the si-NC group had fewer autophagosomes in renal podocytes, with disrupted MAM, higher ER–mitochondria contact levels, and tighter MAM contacts compared to the control. The si-PTPIP51 group showed an increase in autophagosomes, improved MAM, and slightly looser contacts, which were further improved by YHR (Figures 7E,F). Immunofluorescence staining also showed that the immunofluorescence intensity of LC3 was weakened in the si-NC group compared to that in control group, while si-PTPIP51 transfection and YHR treatment enhanced the positive intensity of LC3 in podocytes (Figures 7G,H).

## 4 Discussion

**Figure 8:**
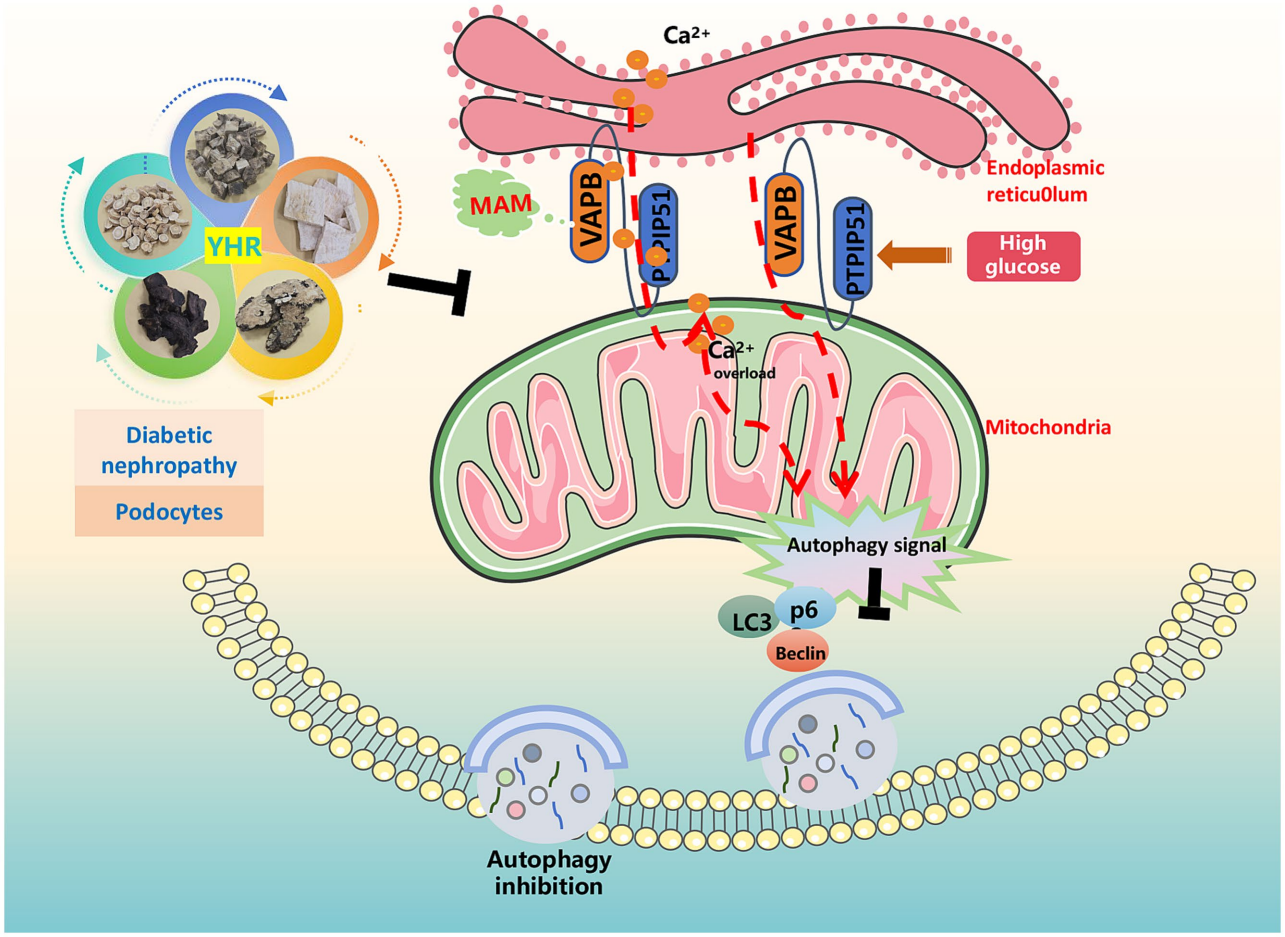
Schematic diagram illustrating the mechanism by which YHR alleviates renal injury in diabetic nephropathy. The figure was partly generated using Servier Medical Art, provided by Servier, licensed under a Creative Commons Attribution 3.0 unported license.

Diabetes, a prevalent chronic metabolic disorder, significantly contributes to the global disease burden. Its most critical complication, DN, is the leading cause of ESRD and renal failure, with a high mortality rate and posing a grave health risk. Identifying effective treatments to mitigate kidney damage and slow DN’s progression is of paramount importance. TCM, with its long-standing history, is emerging as a promising approach in DN treatment (25). In a Taiwanese study of 107,294 DN patients, reduced ESRD and mortality rates were correlated with TCM treatments (26). Another study involving 45 patients indicated that a TCM recipe, Zishen Tongluo, outperformed Benazepril in enhancing metabolism and kidney function in early stage DN patients (27). Recently, interest in exploring the efficacy and mechanisms of Chinese herbal compounds, herbs, and their active ingredients on DN for novel drug development is evident (28). Our research focused on YHR, a clinical formula from our hospital, to investigate its effects on DN recovery and its underlying mechanisms. The results signified that YHR significantly alleviated the damage of the kidney in DN mice and podocytes by HG stimulation, potentially via the regulation of MAM complex VAPB–PTPIP51 to trigger autophagy (Figure 8).

TCM treatment emphasizes a holistic approach, while its advantages of multi-target, low-toxicity treatments are hindered by the complexity of its components, making the identification of active chemicals and mechanisms increasingly vital for research advancements (29). YHR comprises five TCM herbal medicines, with complex chemical compositions. Through UPLC-MS/MS chemical analysis, we mapped YHR’s fingerprint to pinpoint its active compounds, identifying 83 in total, including Isoastragaloside I, Rehmannioside A, Jioglutin A, Puerarin-xyloside II, Genistein, Rehmaglutin B, Formononetin, Biochanin A, Cistanoside A, and others etc. Some studies suggest these compounds as the active components of YHR for DN treatment. Genistein, a primary component in *Radix Puerariae*, has been shown to effectively suppress inflammation in mouse renal tubule cells and reduce lipid peroxidation levels in the plasma and urine of diabetic mice under HG and albumin conditions (30). Formononetin, found in *Astragalus propinquus*, is reported to decrease renal tubular cell apoptosis and mitochondrial damage, alleviate ALB, and ameliorate kidney damage in DN rats (31). Biochanin A exhibits anti-diabetic, antihyperlipidemic, antioxidant properties, and protective effects against DN, potentially by modulating the apoptotic cascade involving TGF-β1, PAR-2, and the NF-κB/NLRP3 axis (32, 33). Isoastragaloside I, a saponin in *Astragalus propinquus*, promotes the differentiation of pancreatic duct organoids into insulin-producing cells (34). Rehmannioside A in *Rehmannia Radix* mitigates HG-induced apoptosis and oxidative stress in renal tubule epithelial cells (35). Collectively, these findings underscore the therapeutic potential of YHR in managing diabetic nephropathy.

Our *in vitro* and *in vivo* studies have also revealed the beneficial effects of YHR on DN-induced kidney injury and HG-induced podocyte damage. These benefits are accompanied with relaxed the MAM contact, reduced mitochondrial calcium transport, and autophagy activation. MAM, a crucial linker between the mitochondria and endoplasmic reticulum, controls calcium homeostasis, mitochondrial function, lipid metabolism, autophagy, and apoptosis. Dysregulation of MAM has been implicated in the onset and progression of diabetes and DN. The stability of MAM structure is consistently affected under conditions of glucose toxicity and insulin resistance (36). Studies have observed disrupted MAM integrity in DN patients, which is correlated with lipid accumulation and kidney impairment (37). Blocking intracellular ER–mitochondria contact has been found to counter DN by limiting aberrant MAM production (38, 39). MAM disruption can also influence ER–mitochondrial Ca2 + transport, and excessive mitochondrial calcium loading resulting in podocyte protein filtration dysfunction is one crucial feature of DN (40). Additionally, Yu et al. (41) have reported that inhibition of MAM-related autophagy could promote the Alzheimer’s disease. In sum, it is suggested that YHR’s therapeutic effect on DN-induced kidney injury involves relaxed MAM contact, reduced mitochondrial calcium overload, and autophagy activation.

In further studies of the HG-activated podocytes, YHR was found to function via autophagy activation through MAM complex VAPB–PTPIP51. Research indicates that enhancing autophagy can protect podocytes and mitigate kidney injury (16). The active chemicals in YHR, including Genistein (42), Biochanin A (43), Cistanoside A (44), Jionoside A1 (45), have also been found to boost autophagy to alleviate damages across a spectrum of diseases. Dai et al. (46) have reported that YHR delayed intervertebral disc degeneration by promoting the formation of Beclin1–VPS34 complex to activate autophagy. Another study also reveals the autophagy regulation effect of YHR in DN, associated with the mTOR/S6K1/LC3 pathway (11). The formation of MAM depends partly on the interaction between MAM complex VABP–PTPIP51, while overexpression of VABP–PTPIP51 leads to the suppression of autophagosome formation (19). Previous studies on VABP–PTPIP51 have primarily focused on its role in aging-related diseases (47). However, in this study, we demonstrate its role in DN where high expression of VABP–PTPIP51 results in autophagy inhibition and podocytes dysfunction. After inhibiting PTPIP51, an increase in the number of autophagosomes was observed, indicating that the autophagic process was activated. More importantly, YHR further activated autophagy, alleviating these abnormal conditions. It is proposed that YHR induces autophagy by modulating the MAM complex VAPB–PTPIP51.

It is necessary to acknowledge the limitations of this study, which serves as a preliminary exploration of YHR’s efficacy and possible mechanisms in treating DN. Further in-depth research is required to ascertain these mechanisms. Additionally, although YHR has been revealed as a TCM for in treating DN by this study, the full characterization of its key pharmacodynamic chemicals remains incomplete, which warrants further exploration. While active compounds Isoastragaloside I is certified by Xu to alleviate diabetes by improving insulin resistance, the contributions of the other components remain to be investigated (48). This gives further indication for our next research.

In summary, we established HG cell and DN mouse models to identify overexpression of VAPB–PTPIP51, alterations in MAM structure, mitochondrial calcium overload, and inhibition of autophagy, alongside kidney and podocyte damages. YHR’s therapeutic impact in DN appears to involve enhancing autophagy, modulating mitochondrial calcium levels through the MAM complex VAPB–PTPIP51. This research aims to elucidate the mechanisms of YHR in treating DN, offering a theoretical foundation for the clinical application of TCM and the development of new drugs for DN therapy.

## Data Availability

The original contributions presented in the study are publicly available. This data can be found here: https://www.jianguoyun.com/p/DYIb1GoQ0NG6DRjZ6foFIAA.

## Supplementary material

TABLE S1 Chemical components of YHR.
