# Supplementary material for: Yiqi Huoxue recipe ameliorates diabetic nephropathy by mediating VAPB–PTPIP51 complex to activate autophagy and regulate MAM contact
Source: Front Nutr. 2025 Nov 3;12:1634555. doi: 10.3389/fnut.2025.1634555 (PMC12620390; doi:10.3389/fnut.2025.1634555)
Supplement: Supplementary file 2 [file Table_2.DOCX]

Supplementary Table 2. Primers designed for Real-Time PCR (mouse)

| Gene name | Forward Primer | Reverse Primer |
| --- | --- | --- |
| PTPIP51 | CCGGGTTGGGACTGTTACTT | TAGTGACGTCTGGCAGTTCC |
| VAPB | ACACGAGCTCAAGTTCCGAG | GCCTTCCTCACCCGAAGTC |
| GAPDH | GGTGAAGGTCGGTGTGAACG | CTCGCTCCTGGAAGATGGTG |
